# Neighborhood Disorder and Obesity-Related Outcomes among Women in Chicago

**DOI:** 10.3390/ijerph15071395

**Published:** 2018-07-03

**Authors:** Stephanie L. Mayne, Angelina Jose, Allison Mo, Lynn Vo, Simona Rachapalli, Hussain Ali, Julia Davis, Kiarri N. Kershaw

**Affiliations:** Department of Preventive Medicine, Northwestern University Feinberg School of Medicine, 680 North Lake Shore Drive Suite 1400, Chicago, IL 60611, USA; angelinajose2020@u.northwestern.edu (A.J.); allisonmo2021@u.northwestern.edu (A.M.); lynnvo2018@u.northwestern.edu (L.V.); simonarachapalli2020@u.northwestern.edu (S.R.); hussainali2019@u.northwestern.edu (H.A.); juliadavis2018@u.northwestern.edu (J.D.); k-kershaw@northwestern.edu (K.N.K.)

**Keywords:** neighborhoods, physical disorder, Google Street View, obesity, physical activity, diet

## Abstract

Neighborhood psychosocial stressors like crime and physical disorder may influence obesity-related outcomes through chronic stress or through adverse effects on health behaviors. Google Street View imagery provides a low-cost, reliable method for auditing neighborhood physical disorder, but few studies have examined associations of Street View-derived physical disorder scores with health outcomes. We used Google Street View to audit measures of physical disorder for residential census blocks from 225 women aged 18–44 enrolled from 4 Chicago neighborhoods. Latent neighborhood physical disorder scores were estimated using an item response theory model and aggregated to the block group level. Block-group level physical disorder scores and rates of police-recorded crime and 311 calls for service requests were linked to participants based on home addresses. Associations were estimated for 6 obesity-related outcomes: body mass index, obesity, total moderate-to-vigorous physical activity, and weekly consumption of sugar-sweetened beverages, fast food, and snacks. Hierarchical regression models estimated cross-sectional associations adjusting for individual sociodemographics and neighborhood poverty. Higher neighborhood physical disorder was associated with greater odds of obesity (OR: 1.43, 95% CI: 1.01, 2.02). Living in a neighborhood with a higher crime rate was associated with an increase in weekly snack consumption of 3.06 (95% CI: 1.59, 4.54).

## 1. Introduction

Physical and social characteristics of neighborhoods are increasingly recognized as important determinants of cardiometabolic health [1]. Repeat exposure to neighborhood physical and social disorder (e.g., graffiti, abandoned buildings, and crime) may trigger an ongoing physiological stress response resulting in dysregulation of neuroendocrine and inflammatory processes [2,3,4], a pathway that has been associated with central adiposity [5]. Residents of disordered neighborhoods may also be less likely to engage in physical activity [6] and more likely to engage in unhealthy behaviors as a coping mechanism [7,8]. Finally, perceptions of neighborhood disorder (which may be influenced by racial stereotyping and stigma) may lead to further economic disinvestment in disadvantaged neighborhoods [9], which can limit access to resources and amenities and may have negative health impacts.

Most prior neighborhood research has utilized administrative data, such as census variables, to characterize neighborhood features [10]. However, census data are collected for administrative purposes and tend to focus on information regarding neighborhood structure/composition. These data sources cannot capture certain neighborhood features that may be relevant to health outcomes, such as physical disorder. One solution to this shortcoming is to use data reported by study participants to characterize neighborhood features that are not available in administrative datasets. However, these data are subject to “same-source bias”, when individuals are reporting on both the outcome and exposure, and the outcome influences how they report the exposure or vice versa [11].

To overcome these limitations, researchers have increasingly conducted systematic social observations using in-person audits of streets or blocks in neighborhoods of interest to more objectively measure neighborhood features [12]. However, in-person audits can be expensive and logistically complicated as auditors must travel to each location and physically walk through the area to record observations. New technologies including Google’s “Street View” feature make it possible to assess these features virtually by viewing high resolution images of neighborhoods of interest and taking a virtual “walk” through the neighborhood [13,14,15]. Prior studies have found Google Street View to be a reliable and cost-effective method of measuring neighborhood characteristics [13,15,16,17], and have used the application to audit neighborhood characteristics including physical disorder [6,13,15,16,17,18,19,20]. While the reliability of this approach has been previously established, few prior studies have examined associations of Google Street View-audited neighborhood physical disorder with health outcomes [6,15].

Our objective was to examine associations of three objective measures of neighborhood physical and social disorder—physical disorder assessed via Google Street View, police-recorded crime rates, and rates of 311 calls for physical disorder-related complaints (e.g., graffiti removal)—with obesity-related outcomes among a sample of women from four neighborhoods in Chicago.

We examined associations with body mass index, obesity, total physical activity, and dietary patterns. We hypothesized that greater physical disorder and higher rates of police-recorded crime and 311 calls would be associated with higher BMI, greater odds of obesity, more frequent consumption of unhealthy foods and sugar-sweetened beverages, and lower levels of physical activity.

## 2. Materials and Methods

### 2.1. Data

#### 2.1.1. Study Population

Data for this study came from the Chicago Healthy Eating Environments and Resources Study (CHEERS), which enrolled 228 women aged 18–44 years old between September 2016 and October 2017. This study was conducted to better understand how women of childbearing age use their environments, their knowledge, and each other to make eating decisions. The study focused on women based on the high burden of obesity in non-Hispanic black and Hispanic women and the fact that women are typically responsible for the food preparation and purchasing for their families. This age group was chosen because it represents a critical period of increased weight due to a multitude of factors including post-pregnancy weight retention and declining muscle mass and muscle strength.

Participants were recruited from four Chicago neighborhoods of varying socioeconomic status using a nonproportional quota sampling approach. Participants were excluded if they did not understand Spanish or English. Recruitment methods included mailings using commercially available address lists, flyers posted in stores located in the four neighborhoods, and presentations to parent organizations at schools in the target neighborhoods. Data collection took place in community centers or libraries in the target neighborhoods, participants’ homes, or our clinic. All participants provided informed consent. The study was approved by the Northwestern Feinberg School of Medicine Institutional Review Board (study number STU00203035).

Participant residential addresses were geocoded to the census block level using ArcMap version 10.5 (Environmental Systems Research Institute, Redlands, CA, USA). Census blocks are typically three or four-sided geographic areas bounded by streets or other physical features (e.g., railroads, bodies of water) and are the smallest units classified by the U.S. Census Bureau. A total of 225 women (99%) had addresses that were able to be geocoded to an address point/street address.

#### 2.1.2. Google Street View Physical Disorder Measure

Neighborhood physical disorder was assessed using Google Earth Pro’s Street View functionality (Google, Inc., Mountain View, CA, USA). Street View is a tool that provides freely available panoramic, high definition street-level imagery captured using cameras mounted on cars. Adjacent cameras take overlapping pictures and images are stitched together to avoid gaps in the imagery and to create a 360-degree view of the street.

We created a physical disorder scale based on previously validated measures [15,21]. We selected nine items from these scales in order to include all indicators we theorized to be relevant to physical disorder (Table 1). Physical disorder items were recorded by raters while taking a virtual walk around each block in Google Earth Pro. Eight raters were trained on using Google Earth and the physical disorder instrument in a two-hour session. Raters completed a set of 10 practice blocks prior to beginning data collection, and coding decisions were discussed to reach consensus. Data collection took place between October 2017 and April 2018. Each block was coded by two raters in order to assess inter-rater reliability. Raters entered Google Earth street view and took a virtual walk around each participant’s block of residence to audit indicators of physical disorder. The unit of data capture was the block face, or one side of a single street segment on the block (range 3–6 block faces in a block, median 4). Google Street View imagery dates for the coded block faces ranged from July 2007 to October 2017, with 85% of block faces having imagery captured in 2015–2016.

Data were recorded and managed using REDCap electronic data capture tools hosted at Northwestern University Feinberg School of Medicine [22]. REDcap is a secure, web-based application for building and managing online data capture for research studies, providing (1) an intuitive interface for validated data entry; (2) audit trails for tracking data manipulation and export procedures; (3) automated export procedures for seamless data downloads to common statistical packages; and (4) procedures for importing data from external sources.

We assessed inter-rater reliability for the full sample of block faces with imagery available in Street View (*N* = 662). We calculated percent agreement between the two raters for each block face and calculated Cohen’s kappa statistics for physical disorder items with prevalence ≥10%. For low prevalence items, the expected chance agreement is inflated and kappas are artificially low, so we did not calculate kappas for items with prevalence <10% [23].

In order to construct a score reflecting a latent level of neighborhood physical disorder, we used an item response theory (IRT) model which estimated the log odds of observing a given physical disorder indicator for a given block face as a function of a latent level of physical disorder. This method has been used previously to combine physical disorder indicators measured using Google Street View into a summary score [21]. The IRT model estimated the parameters of severity (reflecting the level of latent disorder at which there is a 50% probability of observing that item) and discrimination (how accurately the item distinguishes high from low disorder neighborhoods). The model allowed for different discrimination levels between items. We calculated the internal consistency reliability of the IRT model using the formula 1—(1/I), with I indicating the area under the total information curve, as described previously [21]. Block face-level latent physical disorder scores were aggregated to the census block level by taking the average score across all block faces, then aggregated to the block group level by averaging scores from all blocks within a block group (range 1–8 blocks in a block group, median 1) in order to correspond with the other neighborhood measures.

#### 2.1.3. Police-Recorded Crime Rates

Crime data were extracted from the City of Chicago’s Data Portal [24], which published a database of all police-recorded crime occurring in the city limits. We obtained crime data from January to December of 2016. Crimes were categorized according to a previously published classification scheme [25,26] using Illinois Uniform Crime Reporting codes. Crimes were categorized as: assault/battery, homicide, criminal offenses (e.g., robbery, sexual assault, and arson), and incivilities (nonviolent crimes that may indicate neighborhood physical/social disorder such as narcotics, prostitution, or vandalism). We calculated population-normalized past-year rates of police-recorded crime for each census block group in the study. The rate was calculated by dividing the total count of crimes in the block group during 2016 by the total population of the block group based on the 2010 U.S. census, then multiplying by 100 to reflect rates per 100 persons. We examined any crime, as well as individual categories.

#### 2.1.4. Rates of 311 Calls for Physical Disorder-related Complaints

The Data Portal also includes a database of 311 calls, or nonemergency calls requesting city services and information on programs or events within the city of Chicago. Included in the database are 311 calls for three complaints related to neighborhood physical disorder: graffiti removal requests, reports of abandoned vehicles, and reports of abandoned buildings. As described above, we extracted counts of each of the three types of 311 calls for each block group during the year 2016 and calculated population-normalized rates per 100 persons in the block group. We examined total 311 calls, as well as the three separate types of calls.

#### 2.1.5. Outcomes

Body mass index was assessed using in-person measurements of height (in meters, assessed without shoes using a portable stadiometer) and weight (in kilograms, assessed in light clothing). We examined BMI continuously, and dichotomized it to indicate whether participants were obese (defined as BMI ≥ 30 kg/m^2^) or not obese (BMI < 30 kg/m^2^). Physical activity was assessed as self-reported minutes per week spent engaging in moderate and vigorous physical activity. Dietary patterns were assessed as self-reported frequency per week of consuming sugar sweetened beverages (regular sodas and fruit drinks, excluding 100% fruit juice), fast food, and snack foods (chips, candy, ice cream, cake, and cookies).

#### 2.1.6. Covariates

Individual-level covariates included self-reported participant age, race/ethnicity (non-Hispanic white, non-Hispanic black, non-Hispanic other race, Hispanic), and educational attainment (less than a high school degree, a high school degree, some college/associates degree, and bachelor’s degree or higher). We also adjusted for neighborhood poverty, defined as census block group-level percent of the population below the federally-defined poverty level.

### 2.2. Statistical Analysis

We examined the distribution of study outcomes, neighborhood physical and social disorder measures, and covariates using descriptive statistics. The three neighborhood exposures were transformed to z scores by subtracting the mean and dividing by the standard deviation. We then used hierarchical linear regression models with block group random intercepts to estimate cross-sectional associations of a 1-SD higher neighborhood physical disorder score, police-recorded crime rate, and rate of 311 calls with BMI, total moderate and vigorous physical activity, and weekly consumption of SSBs, fast food, and snacks. We used hierarchical logistic regression models to assess associations with obesity. We ran two sets of models, the first adjusted for participant age, race/ethnicity, and education; and the second additionally adjusted for neighborhood poverty. In a sensitivity analysis, we repeated physical disorder models after excluding 2 participants for whom all coded block faces in their census block group had imagery dates earlier than 2015 (corresponding to the start of data collection for CHEERS). All statistical analyses were completed using Stata version 14.2 (StataCorp, College Station, TX, USA).

## 3. Results

### 3.1. Descriptives

Among 225 women, 48% were Hispanic, 15% were non-Hispanic black, 35% were non-Hispanic white, and 3% were non-Hispanic other race (Table 2). Approximately half had a bachelor’s degree or higher (110, 49%). The mean percent of neighborhood population below the poverty level was 17% (SD 12%). Participants lived in a total of 193 census blocks, 121 block groups, and 73 census tracts.

The prevalence of the individual physical disorder items ranged from 4.6% for abandoned vehicles to 31.3% for bars on windows/doors (Table 1). Inter-rater reliability for the physical disorder was calculated on the entire sample. Percent agreement ranged from 75.8% to 93.2% (average 85.1%) and kappas ranged from 0.38 to 0.56 (average 0.47, indicating moderate agreement). Block group-level latent physical disorder scores estimated using the IRT model ranged from −0.68 to 1.20 (mean: 0.11, SD: 0.45). The IRT disorder measure’s internal consistency score was 0.86, suggesting the scale measures a consistent underlying construct. Item difficulties ranged from 1.28 (more common) for graffiti to 3.44 (very rare) for abandoned cars. Item discriminations ranged from 0.46 (weak) for other defaced property to 2.12 (strong) for most buildings in poor/deteriorated condition.

The mean neighborhood police-recorded crime rate was 3.8 per 100 persons in the block group per year (SD: 2.8). The mean rate of 311 calls for graffiti removal, reporting abandoned vehicles, or reporting abandoned buildings was 7.6 per 100 persons in the block group per year (SD: 7.9). Neighborhood variables were moderately correlated, with Spearman correlation coefficients between the three neighborhood disorder measures, as well as neighborhood poverty, ranging from 0.26 (between rate of 311 calls and neighborhood poverty) to 0.53 (between the latent neighborhood physical disorder score and rate of 311 calls).

### 3.2. Regression Models

Table 3 presents results from hierarchical linear and logistic regression models estimating associations of a 1-SD higher neighborhood physical disorder score, crime rate, or rate of 311 calls with the 6 obesity-related outcomes. Higher neighborhood physical disorder was associated with significantly greater odds of obesity upon adjustment for individual-level characteristics and neighborhood poverty (OR 1.43, 95% CI: 1.01, 2.02). Neighborhood physical disorder was not associated with the other outcomes. Rates of 311 calls were not associated with any obesity-related outcomes. Neighborhood crime rates were associated with a significant increase in weekly unhealthy snack consumption of 3.06 times per week (95% CI: 1.59, 4.54) after adjustment for age, race, educational attainment, and neighborhood poverty. Crime rates were not associated with any other outcomes (Table 3). Associations were similar when we examined individual crime categories (homicide, assault/battery, criminal offenses, and incivilities—data not shown). Results were similar in a sensitivity analysis excluding 2 participants who did not have Google Street View imagery from 2015–2016 available for any block faces coded in their census block group (Supplemental Table S1).

## 4. Discussion

In this cross-sectional study of neighborhood physical and social disorder and obesity among women from four Chicago neighborhoods, we found living in a neighborhood with higher physical disorder was associated with higher odds of obesity. In addition, living in a higher crime neighborhood was associated with greater weekly consumption of unhealthy snacks. We found no association between rates of 311 calls for physical disorder-related complaints and obesity-related outcomes. The items in the physical disorder measure used in our study had high percent agreement between raters and moderate kappa values, comparable to what has been found in other studies [13,15,18,21].

Neighborhood social and physical disorder are hypothesized to influence obesity through two pathways. First, these factors may operate directly through a biological stress response pathway. Exposure to crime has been shown to increase psychological distress [27] and may lead to chronic stress [28]. Chronic stress may result in prolonged activation of the sympathetic nervous system and secretion of stress hormones, a process linked with inflammation and greater storage of fat around the abdomen [29]. The second hypothesized pathway is through adversely impacting health behaviors. Fear of crime or signs of physical disorder may deter neighborhood residents from engaging in physical activity near their home, and might promote the adoption of unhealthy eating habits as a method of coping with stress [30].

Prior studies have found mixed results for associations of crime and physical disorder with BMI and physical activity. For BMI, most prior studies have used perceived neighborhood safety/disorder rather than objective measures [30,31,32,33,34,35,36,37,38,39,40]. Many [30,32,33,34,35,36,37,38,39,40], but not all [31,41,42] studies, have found positive associations with BMI or obesity. For physical activity, prior studies have found mixed results [6,25,36,42,43,44,45,46]. For example a recent study among older adults in New York City that used Google Street View audits to ascertain neighborhood physical disorder found higher levels of disorder to be associated with lower physical activity at baseline, but not with changes in disorder over time [6].

Prior studies have rarely examined whether neighborhood stressors are associated with unhealthy dietary patterns. Negative emotions have been found to increase consumption of energy-dense foods high in sugar, salt, and saturated fat [47,48]. As such, consumption of high sugar or fat foods as a coping mechanism is a hypothesized behavioral mechanism through which living in a stressful environment might increase the risk of obesity [30]. In a study of children in Birmingham, Alabama, Keita et al. found higher perceived neighborhood disorder to be marginally associated with higher energy intake and higher sodium intake [49]. In addition, poor self-reported diet quality was found to mediate associations of psychological distress and obesity (which in turn mediated associations of neighborhood disorder with obesity) in a study among Texas adults [30]. In our study, higher crime rates were associated with higher levels of consumption of snacks (chips, candy, ice cream, cookies, and cake) among adult women, but not associated with sugar-sweetened beverage or fast food consumption. These results provide some support for the potential for stressful neighborhood environments to influence eating behaviors, but further work is needed in this area.

Objective neighborhood social environment measures, such as those obtained from systematic social observations or police-recorded crime rates, avoid one of the potential pitfalls of defining neighborhood characteristics based on participant report: same source bias. Same source bias may occur when participants self-report both an exposure and an outcome, and their perceived exposure influences how they perceive the outcome or vice versa [12]. However, it is possible that these objective measures do not align well with how individuals actually perceive their environment, and that the perception is more influential for health outcomes. Several prior studies that examined both individual-level perceptions of the neighborhood environment and more objective measures (e.g., police-recorded crime or aggregated, neighborhood-level measures) have found stronger associations between individual perceptions and health outcomes including obesity [50], depression [51], and blood pressure [52]. In addition, a study comparing resident perceptions to Google Street View audits in five European urban areas found relatively low agreement between the two types of measures, particularly related to aesthetics such as litter and graffiti [53]. Although an objective measure of neighborhood physical disorder was associated with obesity in our study, lack of correspondence between perceived and objective neighborhood environments may be a potential explanation for the largely null findings for other outcomes in our study. Further work is needed to characterize the relative importance of objective versus perceived measures of the neighborhood social environment for health outcomes.

This study was subject to several limitations. First, data were cross-sectional and could not establish temporality or evaluate changes in neighborhood characteristics over time. We used Google Street View imagery at one time point to define neighborhood disorder for each block face, and not all imagery was captured at the same time. In addition, we did not assess participants’ perceptions of neighborhood safety or disorder, which might differ from the objectively captured measures used in this study as noted above. As the focus of our analysis was on the neighborhood social environment, we did not collect information on other neighborhood characteristics such as availability of healthy food retail, access to parks, or physical activity resources. Our sample included women recruited from four Chicago neighborhoods and nearly half of participants had a bachelor’s degree or higher, so results may not generalize to other populations. In addition, the small sample size and relatively limited variability in neighborhood exposures may have limited our ability to detect weaker associations.

## 5. Conclusions

Among a cohort of women in Chicago, living in a neighborhood with higher physical disorder (measured using virtual audit) was associated with greater odds of obesity. In addition, living in a higher crime neighborhood was associated with greater consumption of unhealthy snacks. Rates of 311 calls were not associated with obesity-related outcomes.

## Figures and Tables

**Table 1:** Neighborhood Physical Disorder Items and Inter-Rater Reliability for Block Faces Audited using Google Street View ^1,2^.

List of Items	Categorization	Prevalence	% Agreement	Kappa
Trash/garbage	Heavy/moderate (1) vs. light/none (0)	22.8%	80.2%	0.44
Abandoned vehicle	Yes (1) vs. no (0)	4.6%	93.2%	--
Graffiti	Yes (1) vs. no (0)	23.1%	82.4%	0.50
Graffiti scrubbed or painted over	Yes (1) vs. no (0)	19.1%	84.9%	0.51
Other defaced property	Yes (1) vs. no (0)	28.4%	75.8%	0.41
Bars on windows/doors	Yes (1) vs. no (0)	31.3%	80.9%	0.56
Abandoned/boarded up buildings	Yes (1) vs. no (0)	8.6%	89.9%	--
Building condition	Poorly deteriorated (1) vs. well-kept/moderate (0)	12.0%	86.8%	0.38
Vacant lots	Yes (1) vs. no (0)	7.8%	91.4%	--

^1^ Two raters coded each block face (*n* = 662 block faces from 193 census blocks) and all block faces were used to calculate percent agreement and kappa statistics. ^2^ Kappa statistics were not calculated for items with prevalence <10%.

**Table 2:** Characteristics of Study Population.

Characteristic	*N* (%) or Mean (SD)
Total *N*	225
Socio-demographics:	
Age—Mean (SD)	33.9 (7.0)
Race/Ethnicity—*N* (%)	
Hispanic	108 (48.0)
Non-Hispanic White	78 (34.7)
Non-Hispanic Black	33 (14.7)
Non-Hispanic, Other Race	6 (2.6)
Education—*N* (%)	
<High School degree	36 (16.0)
High School degree	44 (19.6)
Some College/Technical School/Associate’s Degree	35 (15.5)
Bachelor’s Degree or Higher	110 (48.9)
Neighborhood Poverty—Mean (SD) ^1^	17.2 (11.9)
Outcomes:	
Body mass index—Mean (SD)	29.0 (7.1)
Obese (BMI ≥ 30)—*N* (%)	79 (35.3)
Minutes per week of moderate-to-vigorous physical activity—Mean (SD) ^2^	252.3 (412.2)
Sugar sweetened beverage consumption (times per week)—Mean (SD) ^2^	3.1 (6.3)
Fast food consumption (times per week)—Mean (SD) ^2^	1.2 (4.1)
Snack (chips, candy, ice cream, cake, cookies) consumption (times per week)—Mean (SD) ^2^	7.0 (11.1)
Neighborhood Exposures:	
Google Street View Physical Disorder ^3^	0.11 (0.45)
Police-Recorded Crime Rate ^4^	3.8 (2.8)
Rate of 311 calls ^5^	7.6 (7.9)

^1^ Percent of block group population with household incomes below the federal poverty level; ^2^ Based on self-report; ^3^ Measured using virtual neighborhood audit of Google Street View imagery and aggregated to the census block group level. Physical disorder items were used to estimate a latent physical disorder score using an Item Response Theory (IRT) model. Block group-level scores ranged from −0.68 to 1.20; ^4^ Per 100 block group population per year. Crimes included homicide, assault/battery, criminal offenses (e.g., robbery, sexual assault, and arson), and incivilities (e.g., vandalism, narcotics, and weapons violations). ^5^ Per 100 block group population per year. 311 calls are non-emergency calls requesting services or information from the city. We included only 311 calls related to physical disorder: requests for graffiti removal, reporting an abandoned vehicle, or reporting an abandoned building.

**Table 3:** Associations of neighborhood physical and social disorder with obesity-related outcomes ^1^.

Outcome	Model 1	Model 2
	Beta (95% CI)	Beta (95% CI)
Associations with Body Mass Index (kg/m^2^)		
Physical disorder score	0.72 (−0.23, 1.68)	0.61 (−0.35, 1.58)
Police-recorded crime rate	0.36 (−0.51, 1.24)	0.13 (−0.78, 1.04)
Rate of 311 calls	−0.02 (−0.92, 0.89)	−0.02 (−0.92, 0.88)
Associations with Total Physical Activity ^2^		
Physical disorder score	49.1 (−10.4, 108.7)	52.7 (−7.2, 112.7)
Police-recorded crime rate	−14.0 (−68.4, 40.4)	−9.1 (−66.1, 47.9)
Rate of 311 calls	−22.5 (−78.9, 33.9)	−22.4 (−78.8, 33.9)
Associations with Weekly Sugar-sweetened Beverage Consumption ^3^		
Physical disorder score	0.00 (−0.86, 0.87)	0.05 (−0.82, 0.92)
Police-recorded crime rate	0.59 (−0.19, 1.37)	0.76 (−0.05, 1.58)
Rate of 311 calls	−0.16 (−0.98, 0.65)	−0.16 (−0.98, 0.65)
Associations with Weekly Fast Food Consumption		
Physical disorder score	0.12 (−0.47, 0.71)	0.16 (−0.43, 0.75)
Police-recorded crime rate	−0.05 (−0.59, 0.48)	0.04 (−0.52, 0.60)
Rate of 311 calls	−0.14 (−0.70, 0.41)	−0.14 (−0.69, 0.41)
Associations with Weekly Snack Consumption ^4^		
Physical disorder score	−0.31 (−1.91, 1.29)	−0.27 (−1.88, 1.35)
Police-recorded crime rate	2.69 (1.28, 4.10) *	3.06 (1.59, 4.54) *
Rate of 311 calls	−0.51 (−2.02, 1.00)	−0.51 (−2.02, 1.00)
	**Odds Ratio (95% CI)**	**Odds Ratio (95% CI)**
Associations with Obesity ^5^		
Physical disorder score	1.40 (0.99, 1.98)	1.43 (1.01, 2.02) *
Police-recorded crime rate	1.03 (0.76, 1.41)	1.09 (0.79, 1.51)
Rate of 311 calls	1.22 (0.88, 1.67)	1.21 (0.88, 1.67)

* *p* < 0.05; ^1^ From hierarchical linear regression models with block group random intercepts. Associations are for a standard deviation higher neighborhood score/rate. Model 1 adjusted for participant age, race, and educational attainment. Additionally, Model 2 adjusted for block group percent of households below the poverty level. Note- 1 participant was excluded from models for body mass index and obesity due to an invalid height (*n* = 224 for those models); ^2^ Total minutes per week of moderate and vigorous physical activity (self-reported); ^3^ Sugar-sweetened beverages (SSBs) included regular sodas and fruit drinks (excluding 100% fruit juice); ^4^ Snacks included chips, candy, ice cream, cake, and cookies; ^5^ Obesity defined as ≥30 kg/m^2^.

## Supplementary Materials

The following are available online at http://www.mdpi.com/1660-4601/15/7/1395/s1, Table S1: Associations of Neighborhood Physical Disorder with Obesity-Related Outcomes ^1^ Excluding 2 Participants for Whom All Google Street View Imagery was Earlier Than 2015 (*n* = 223 participants)
