# Supplementary material for: Neighborhood Disorder and Obesity-Related Outcomes among Women in Chicago
Source: Int J Environ Res Public Health. 2018 Jul 3;15(7):1395. doi: 10.3390/ijerph15071395 (PMC6069019; doi:10.3390/ijerph15071395)
Supplement: Supplementary file 1 [file ijerph-15-01395-s001.docx]

**Table S1.** Associations of Neighborhood Physical Disorder with Obesity-Related Outcomes ^1^ Excluding 2 Participants for Whom All Google Street View Imagery was Earlier Than 2015 (*n* = 223 participants)

|  | Model 1 | Model 2 |
| --- | --- | --- |
| Outcome | **Beta (95% CI)** | **Beta (95% CI)** |
|  | | |
| Body Mass Index (kg/m^2^) | 0.71 (−0.26, 1.67) | 0.59 (−0.38, 1.56) |
| Total Physical Activity ^2^ | 49.0 (−11.0, 109.0) | 52.9 (−7.6, 113.3) |
| Weekly Sugar-sweetened Beverage Consumption ^3^ | 0.00 (−0.87, 0.87) | 0.05 (−0.82, 0.93) |
| Weekly Fast Food Consumption | 0.11 (−0.48, 0.71) | 0.16 (−0.44, 0.76) |
| Weekly Snack Consumption ^4^ | −0.33 (−1.94, 1.28) | −0.29 (−1.91, 1.34) |
|  | **Odds Ratio (95% CI)** | **Odds Ratio (95% CI)** |
| Obesity ^5^ | 1.39 (0.98, 1.97) | 1.42 (1.00, 2.01) |

* *p* < 0.05; ^1^ From hierarchical linear regression models with block group random intercepts. Associations are for a standard deviation higher latent neighborhood physical disorder score. Model 1 adjusted for participant age, race, and educational attainment; Model 2 additionally adjusted for block group percent of households below the poverty level. Note- 1 participant was excluded from models for body mass index and obesity due to an invalid height (*n* = 222 for those models); ^2^ Total minutes per week of moderate and vigorous physical activity (self-reported); ^3^ Sugar-sweetened beverages (SSBs) included regular sodas and fruit drinks (excluding 100% fruit juice); ^4^ Snacks included chips, candy, ice cream, cake, and cookies; ^5^ Obesity defined as ≥30 kg/m^2.^
